# Supplementary material for: Homeostatic maintenance and age-related functional decline in the Drosophila ear
Source: Sci Rep. 2020 May 4;10:7431. doi: 10.1038/s41598-020-64498-z (PMC7198581; doi:10.1038/s41598-020-64498-z)
Supplement: Supplementary file 1 — Supplementary Information. [file 41598_2020_64498_MOESM1_ESM.docx]

**Homeostatic maintenance and age-related functional decline in the *Drosophila* ear**

Alyona Keder^1^, Camille Tardieu^1^, Liza Malong^1^, Anastasia Filia^2^, Assel Kashkenbayeva^1^, Fay Newton^3^, Marcos Georgiades^1^, Jonathan E. Gale^1^, Michael Lovett^2^, Andrew P. Jarman^3^ & Joerg T. Albert^1,4,5,6,7^*

**Affiliations:**

^1^Ear Institute, University College London, 332 Gray’s Inn Road, London WC1X 8EE, UK

^2^National Heart and Lung Institute, Imperial College London, Guy Scadding Building, Dovehouse Street, London, SW3 6LY, UK

^3^Centre for Discovery Brain Sciences, Edinburgh Medical School, University of Edinburgh, Edinburgh EH8 9XD, Scotland, UK

^4^Centre for Mathematics and Physics in the Life Sciences and Experimental Biology (CoMPLEX), University College London, Gower Street, London WC1E 6BT, UK

^5^The Francis Crick Institute, 1 Midland Road, London NW1 1AT, UK

^6^Department of Cell and Developmental Biology, University College London, Gower Street, London WC1E 6DE, UK

^7^Lead Contact

*Correspondence to: [joerg.albert@ucl.ac.uk](mailto:joerg.albert@ucl.ac.uk)

**Supplementary Figures**


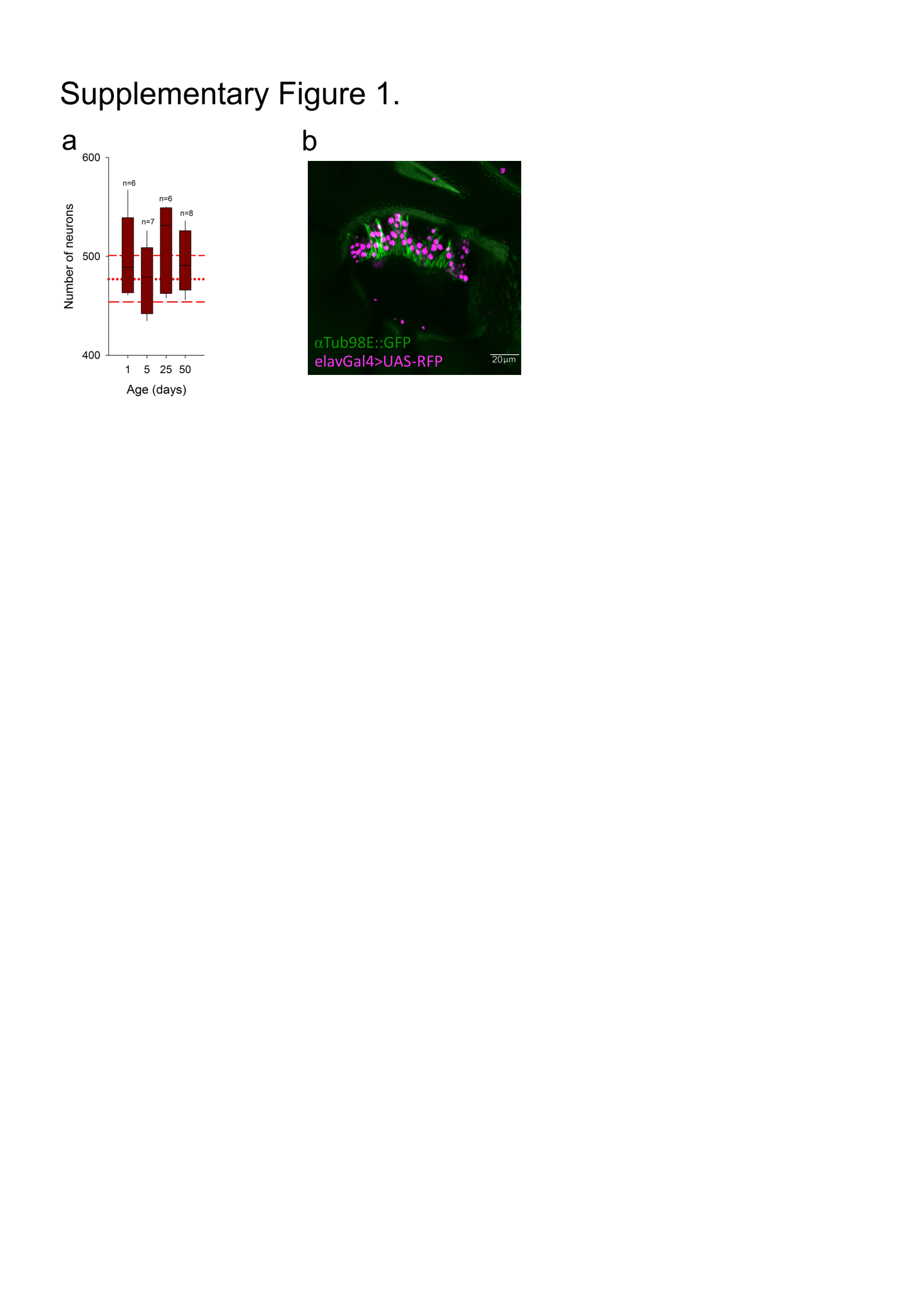


**Supplementary Figure 1. JO neuronal count across the life course.** (a) The number of JO neurons does not change between days 1, 5, 25 and 50. From day 1 to day 50 – i.e. during the time of homeostatic equilibrium – neuronal numbers stay constant. Dotted red line indicates previously published number of neurons (477±24; ^1^), dashed lines indicate standard deviations. (b) Whole-mount preparation of JO expressing the neuronal marker Elav (magenta) and support cell marker αTub85E (green). Whole-mount imaging of JO was used to count JO neurons at different ages.

**
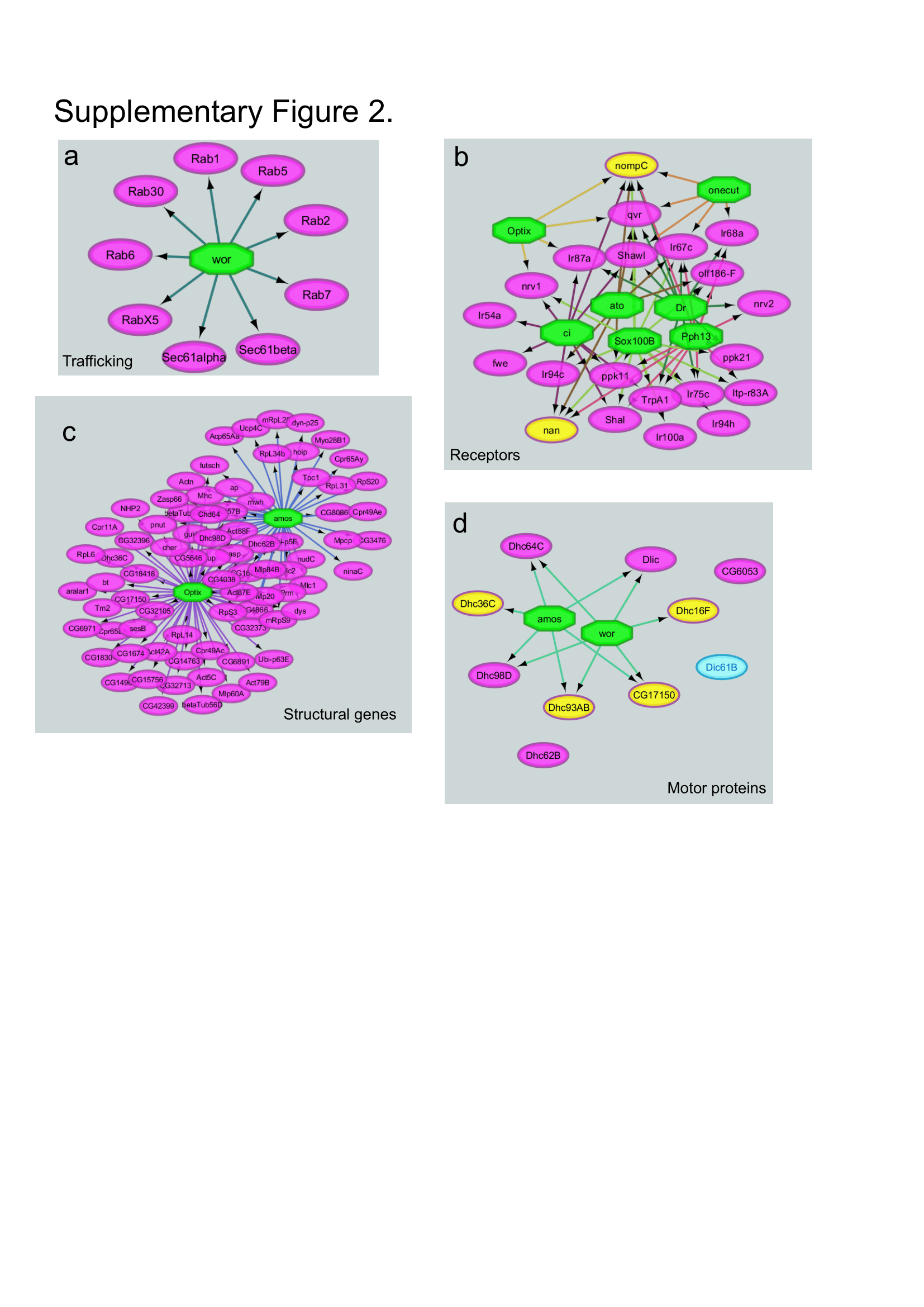
**

**Supplementary Figure 2. Prediction of the downstream regulon using *i*Regulon.**

(a) Trafficking genes predicted downstream of *wor*. (b) Receptors (and ion channels), including NompC and Nan (shown in yellow) are predicted downstream of several transcription factors, including onecut, *Optix*, *ci*, *Sox110B*, *Pph13*, *ato* and *Dr*. (c) Structural genes are predicted to be largely downstream of Amos and Wor. (d) Motor proteins belonging to the dynein family, are predicted downstream of *amos* and *wor*; this also includes dyneins previously identified in JO (shown in yellow) ^2,3^.

**
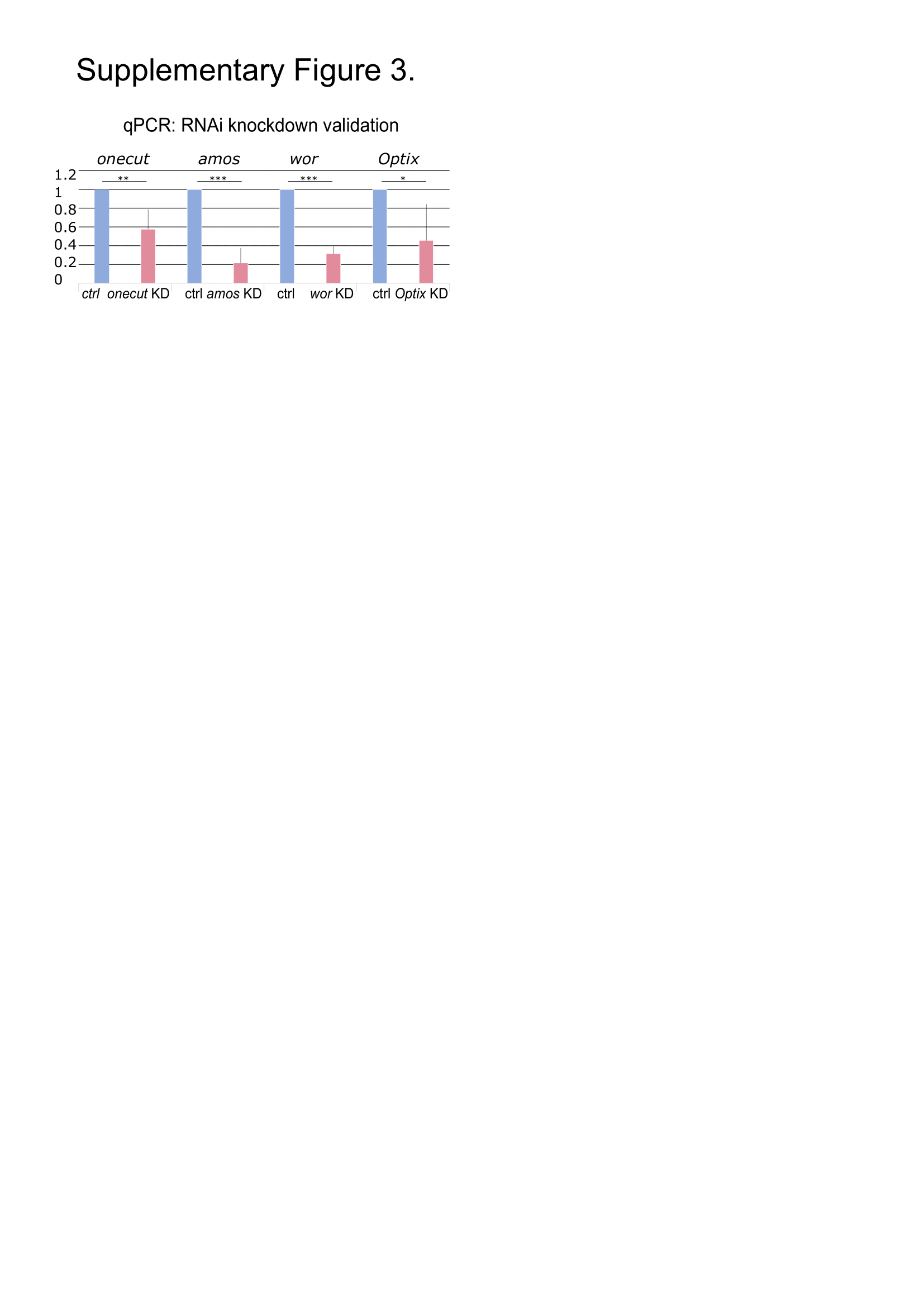
**

**Supplementary Figure 3. RNAi knockdown validation using RT-qPCR.**

Transcription factor (TF) expression changes after their respective RNAi knockdowns. All the knockdowns show significant reduction of the respective TF expression (n=3, * p<0.05, **p<0.01, ***p<0.001).

**
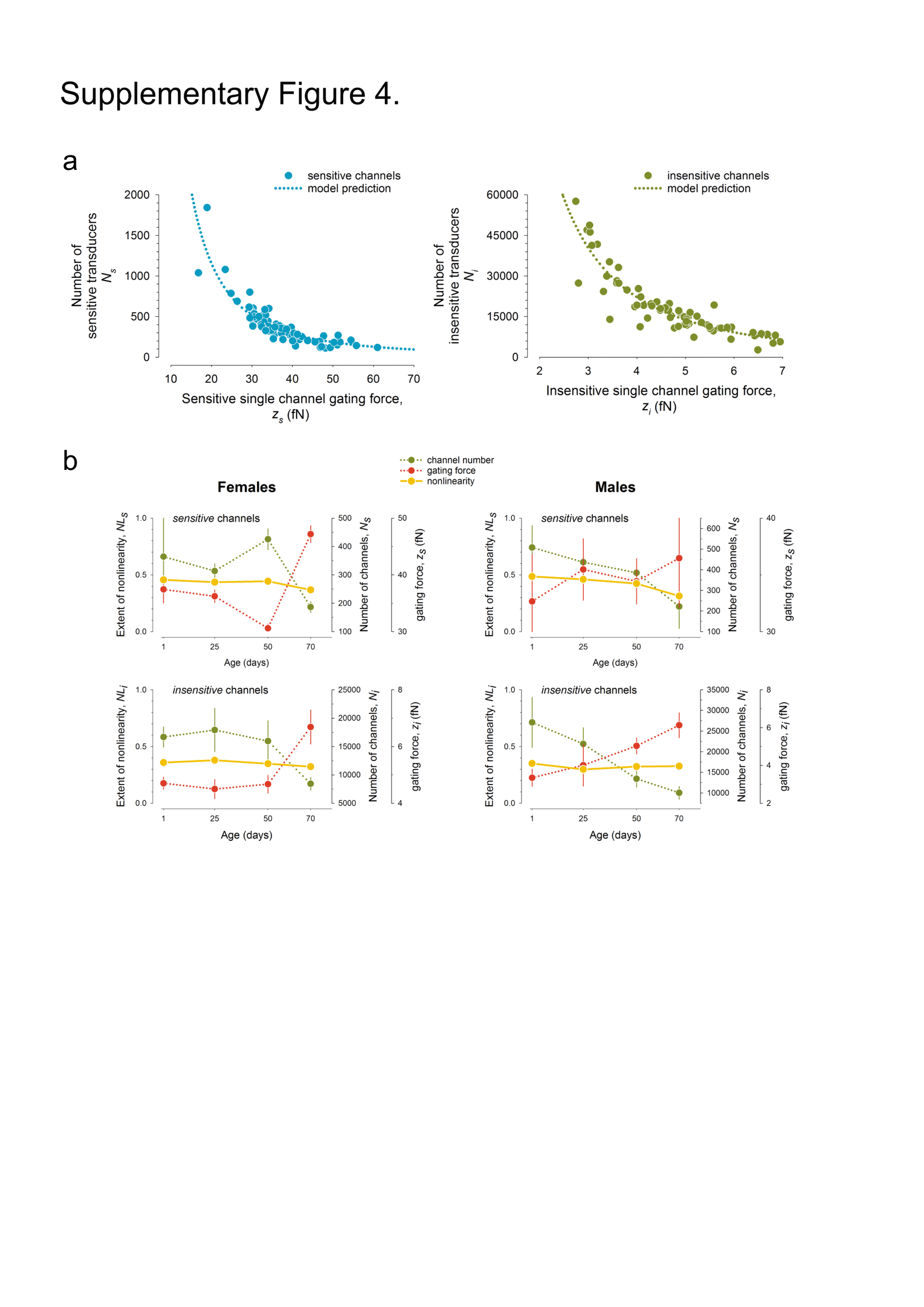
**

**Supplementary Figure 4. Homeostatic link between numbers and gating forces of transducer channels preserves sound receiver nonlinearity.** (a) For both the sensitive (left, blue symbols) and the insensitive (right, green symbols) population of transducers, the relation between channel number (N) and single channel gating force (z) follows a simple model (dashed line), which assumes a constant contribution of the respective transducer population to the overall receiver nonlinearity (corresponding to N~z^-2^, see also ^4^). [Summary data, pooled from all ages and both sexes]. (b) Shown are the numbers (green) and gating forces (red) for sensitive (upper) and insensitive (lower) transducer channels of the individual ages tested, for both females (left) and males (right). Note the age-related changes in both channel numbers and gating forces and the constancy of their resulting contribution to the sound receiver’s extent of nonlinearity (yellow).

**
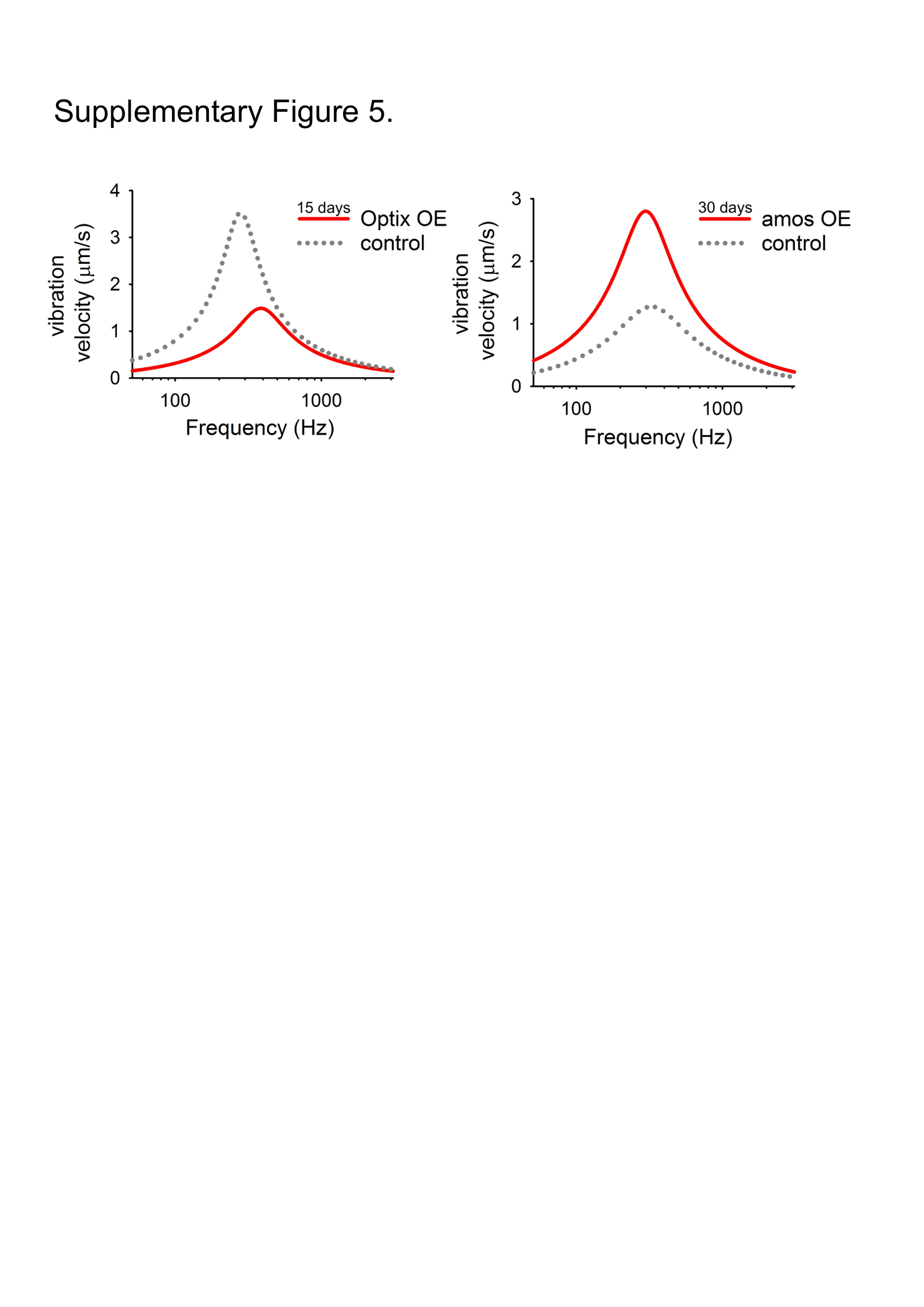
**

**Supplementary Figure 5. Adult-specific overexpression of homeostatic regulators.** (left) Over-expressions of *Optix* for 15 days and (right) *amos* for 30 days revert their respective knock-down phenotypes (compare to the Figure 4a).

**
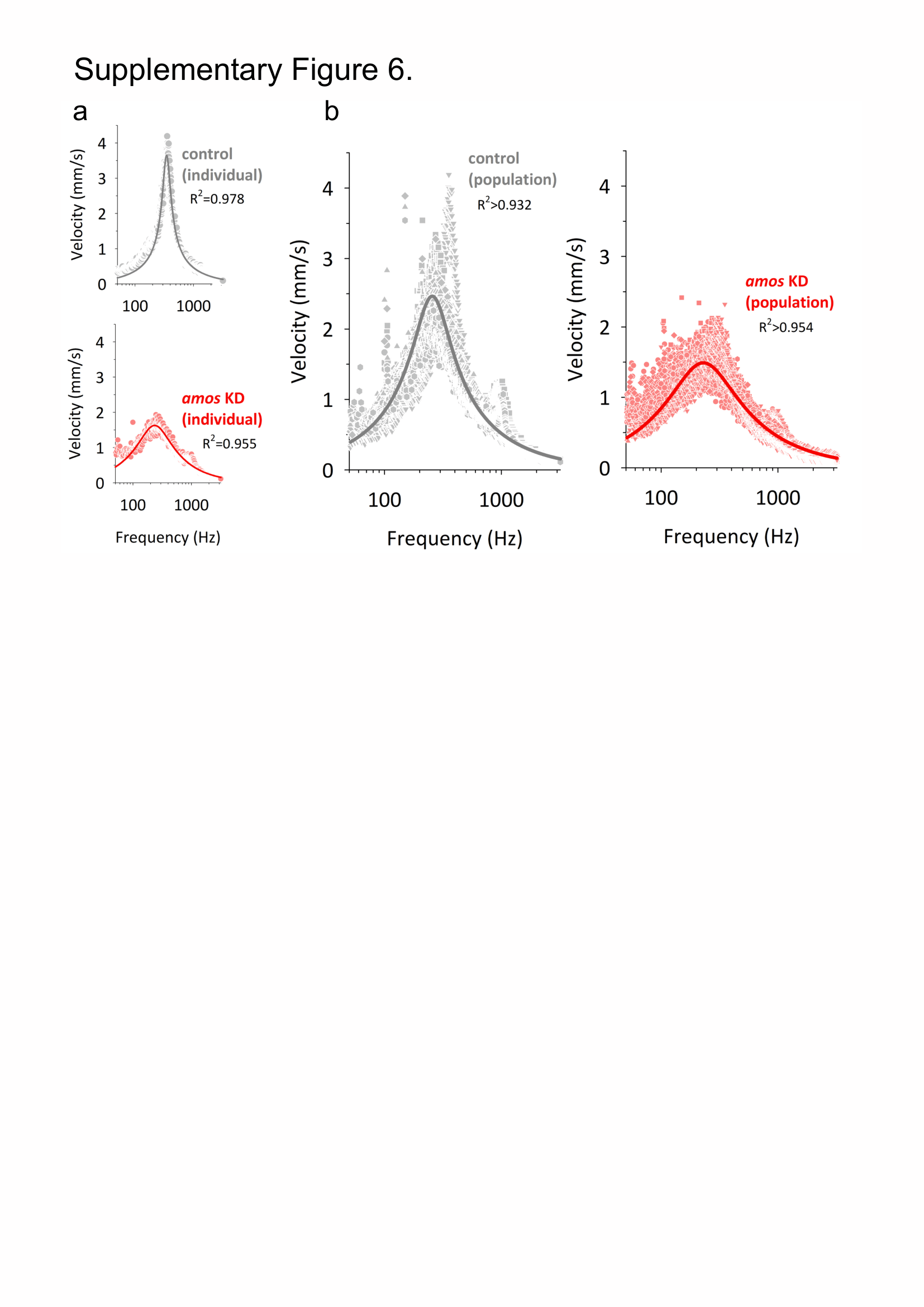
**

**Supplementary Figure 6. Free fluctuation fit procedure.** (a) Vibration velocity data (symbols) and simple harmonic oscillator fits (lines) for a single control (grey, top) and a single *amos* (red, bottom) knockdown (R^2^ values shown); (b) population of vibration velocity raw data (symbols) and median fit (line). Median fits were calculated and replotted from the medians of all individual fit parameters. R^2^ values indicate the lowest value observed.


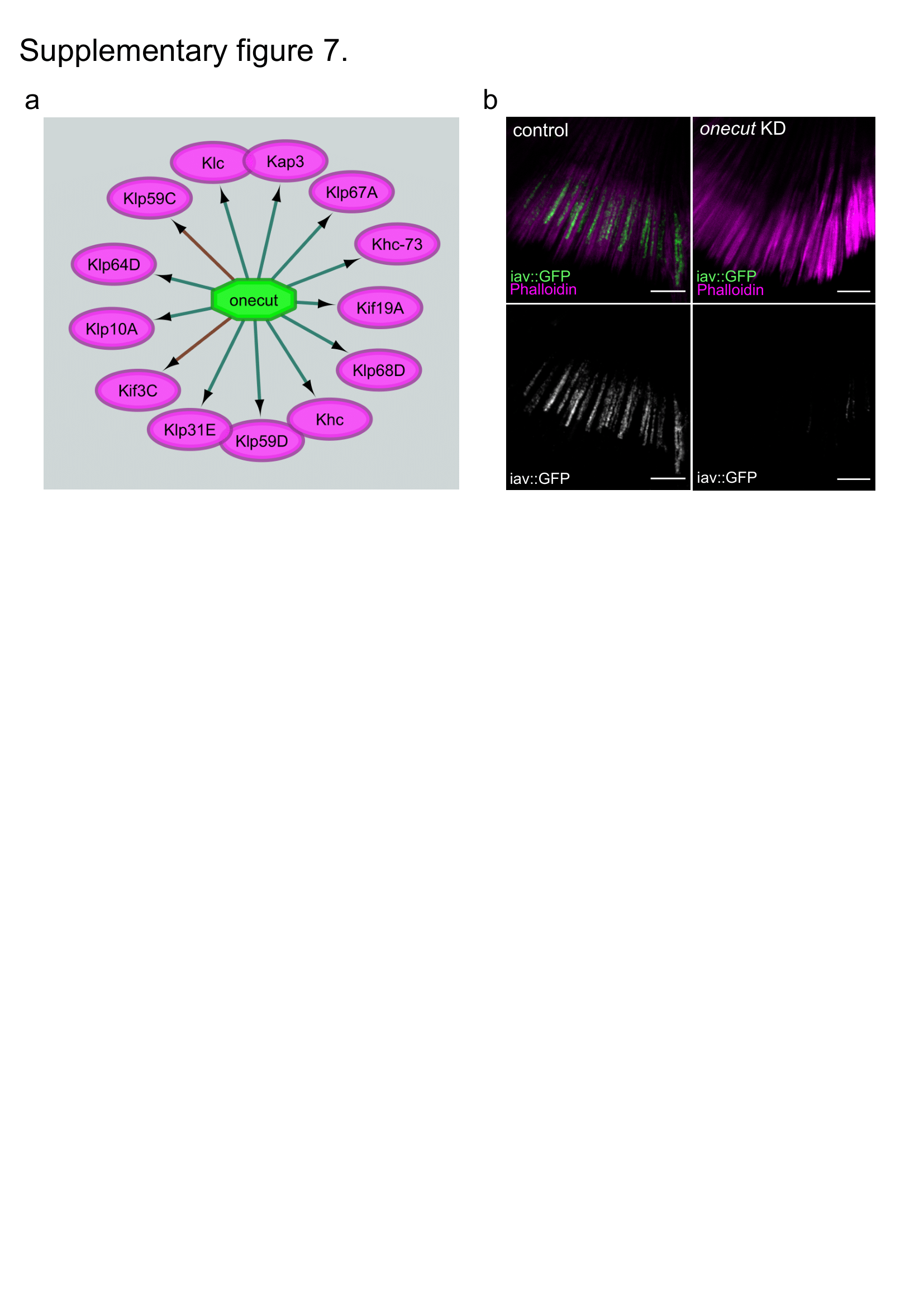


**Supplementary Figure 7. Onecut is required for ion channel transport**

(a) 13 out of 17 kinesin subunits (key components of anterograde ciliary transport) are predicted to be regulated by Onecut; (b) Top panels: (left) Expression of Inactive::GFP (Iav::GFP, green) in the apical cilia of JO neurons in control flies; (right) Iav::GFP is absent in the cilia of Onecut KD flies. Actin-rich scolopale rods are stained with phalloidin (magenta). Lower panels: Iav::GFP channel alone for higher clarity. Scale bars = 5 µm.

**Supplementary Table Legends**

**Supplementary Table 1. *Drosophila* auditory mechanics across the life course.**

Three principal parameters of sound receiver function were assessed in Canton-S and Oregon-R flies at different ages (days 1, 5, 10, 25, 50, 60 and 70): (i) *f_0_ ,* the receivers’ best frequency [in Hz]; (ii) the receivers’ tuning sharpness or ‘quality factor’ *Q* [dimensionless] and (iii) the receivers’ energy gain [in *k_B_T*]*.* Pair-wise student t-tests or Mann-Whitney (MWUS) tests were used to assess statistical significance (choice of test depending on data distribution). Significant changes are highlighted in **bold**. Canton-S (K) and Canton-S (G) are two other Canton-S lines kindly provided to us by Azusa Kamikouchi and Stephen Goodwin, respectively. Both males and females were measured, except for Oregon-R, where only females were measured.

**Supplementary Table 2.** **JO RNA-Seq transcriptomic data across the lifespan for both males and females (see attached excel spreadsheet).**

Flybase ID numbers, gene annotated names, gene-ontological description and the genes’ chromosomal positon are shown for all genes detected in the RNAseq data. Average normalised expression counts are shown in the “baseMean” column, log2Fold changes (log2FC) are shown in pair-wise comparisons between ages of day 50 to day 1 (“log2FC_d50m_d1m”), day 25 and day 1 (“log2FC_d25m_d1m”), etc. *p*-adjusted values are shown in the column “padj”. The analysis was done by using ANOVA. Data is shown for both males (“m”) and females (“f”). Raw counts are shown for each gene at the end of the table, where m1A stands for day1 male replicate number 1, f5B – for female day 5 replicate 2.

**Supplementary Table 3. Age-variable transcriptome of JO (see attached excel spreadsheet).**

Three comparisons were performed of the day 1 to day 5, day 5 to day25 and day 25 to day 50 for genes that were changing in both males and females. The analysis was done by using ANOVA.

Flybase ID numbers, gene annotated names, gene-ontological description, mouse homologues and the gene positon in the chromosomes are shown for all genes of the JO RNAseq data set. Average normalised expression counts are shown in the “baseMean” column. Inclusion criteria were: Log2Fold-Change at least 1.5 times with False Discovery Rate (FDR) of 10%, at least in one of the three comparisons. Signs indicate whether a respective gene was downregulated (negative) or upregulated (positive) for the respective age pair.

**Supplementary Table 4. Summary list of age-variable JO genes identified through RNA-Seq-based transcriptomics (see attached excel spreadsheet).**

JO genes that are either up- or down-regulated in between two age pairs. Age pair 1: (i) day 5 and day 25; age pair 2: (ii) day 25 and day 50. JO genes with significant expression changes are highlighted in red (up-regulated) or blue (down-regulated). Example: genes that are upregulated from day 1 to day 5 with a log2fold change between 2 and 4 are shown as “day 1to5 log2fold 2-4” and highlighted in red. Genes that are down-regulated from day 25 to day 50 with a log2fold change between -1 and -2 are shown as “day25to50 log2fold -2 to-1” and highlighted in blue.

**Supplementary Table 5. 37 iRegulon predicted transcription factors (TFs) expressed in the *Drosophila* JO (see attached excel spreadsheet).** Genes that change their expression in one of the two age comparisons in one or both sexes are shown in bold type. The 19 genes functionally probed in our study are highlighted in light orange. ‘Avg exp’ stands for ‘Average Expression’. Human orthologue genes that are expressed in the adult human inner ear (from Schrauwen and colleagues^5^) are shown in red, the genes that are expressed in the retina (from the study of Ratnapriya and colleagues^6^) are shown in the corresponding column and mice orthologues that are expressed in the mouse inner ear (from MGI database) are show in red in the corresponding column.

**Supplementary Table 6.** **Auditory mechanics after overexpression (OE) or knockdown (KD) of transcriptional regulators.**

Adult-specific manipulation of transcriptional regulators was conducted by using the temperature sensitive repressor tubulin-Gal80[ts] together with the NP0761 Gal4 driver, which drives expression in all JO neurons. Knock-down (KD) or overexpression (OE) of regulators was achieved by using UAS-RNAi or UAS cDNA constructs, respectively. KD or OE constructs were driven for 15 and/or 30 days at 30^0^C and then hearing was assessed through free fluctuation analysis of unstimulated sound receivers as described previously^7^. Three principal parameters of sound receiver function were assessed: (i) f0, the receivers’ best frequency [in Hz]; (ii) the receivers’ tuning sharpness or ‘quality factor’ Q [dimensionless] and (iii) the receivers’ energy gain [in kBT]. Pair-wise student t-tests or Mann-Whitney (MWUS) tests were used to assess statistical significance (choice of test depending on data distribution; significant changes in **bold**). Both males and females were measured.

**Supplementary references**

1 Kamikouchi, A., Shimada, T. & Ito, K. Comprehensive classification of the auditory sensory projections in the brain of the fruit fly *Drosophila* melanogaster. *J Comp Neurol* **499**, 317-356, doi:10.1002/cne.21075 (2006).

2 Karak, S. *et al.* Diverse Roles of Axonemal Dyneins in Drosophila Auditory Neuron Function and Mechanical Amplification in Hearing. *Scientific Reports* **5**, doi:10.1038/srep17085 (2015).

3 Senthilan, P. R. *et al.* Drosophila auditory organ genes and genetic hearing defects. *Cell* **150**, 1042-1054, doi:10.1016/j.cell.2012.06.043 (2012).

4 Albert, J. T., Nadrowski, B. & Göpfert, M. C. Mechanical signatures of transducer gating in the *Drosophila* ear. *Curr Biol* **17**, 1000-1006, doi:10.1016/j.cub.2007.05.004 (2007).

5 Schrauwen, I. *et al.* A comprehensive catalogue of the coding and non-coding transcripts of the human inner ear. *Hear Res* **333**, 266-274, doi:10.1016/j.heares.2015.08.013 (2016).

6 Ratnapriya, R. *et al.* Retinal transcriptome and eQTL analyses identify genes associated with age-related macular degeneration. *Nature genetics* **51**, 606-610, doi:10.1038/s41588-019-0351-9 (2019).

7 Effertz, T., Nadrowski, B., Piepenbrock, D., Albert, J. T. & Göpfert, M. C. Direct gating and mechanical integrity of Drosophila auditory transducers require TRPN1. *Nature Neuroscience* **15**, 1198-1200, doi:<http://dx.doi.org/10.1038/nn.3175> (2012).
