# Supplementary material for: Homeostatic maintenance and age-related functional decline in the Drosophila ear
Source: Sci Rep. 2020 May 4;10:7431. doi: 10.1038/s41598-020-64498-z (PMC7198581; doi:10.1038/s41598-020-64498-z)
Supplement: Supplementary file 2 — Supplementary Table 1. [file 41598_2020_64498_MOESM2_ESM.docx]

| Genotype | age | sex | Parameter | N | Mean | Median | StDev | StErr | p-value |
| --- | --- | --- | --- | --- | --- | --- | --- | --- | --- |
| CantonS | day1 | male | f0 [Hz] | 18 | 229.63 | 217.41 | 74.29 | 17.51 | -- |
|  |  |  | Q |  | 2.81 | 2.21 | 2.04 | 0.48 | -- |
|  |  |  | Energy [kBT] |  | 23.15 | 20.17 | 8.89 | 2.09 | -- |
|  |  | female | f0 [Hz] | 17 | 197.26 | 190.25 | 45.31 | 10.99 | -- |
|  |  |  | Q |  | 2.30 | 2.00 | 0.99 | 0.24 | -- |
|  |  |  | Energy [kBT] |  | 20.69 | 18.45 | 8.31 | 2.02 | -- |
|  | **day5** | **male** | **f0 [Hz]** | **13** | **262.61** | **264.72** | **56.09** | **15.56** | **0.032 (MWUS)** |
|  |  |  | Q |  | 3.03 | 2.94 | 1.33 | 0.37 | 0.389 |
|  |  |  | **Energy [kBT]** |  | **17.27** | **16.37** | **8.28** | **2.30** | **0.048 (MWUS)** |
|  |  | **female** | **f0 [Hz]** | **8** | **238.43** | **232.90** | **29.47** | **10.42** | **0.021 (MWUS)** |
|  |  |  | Q |  | 2.62 | 2.37 | 1.07 | 0.38 | 0.432 |
|  |  |  | Energy [kBT] |  | 14.54 | 13.07 | 6.34 | 2.24 | 0.0774 |
|  | **day10** | **male** | f0 [Hz] | 6 | 206.76 | 193.86 | 43.98 | 17.95 | 0.117 |
|  |  |  | **Q** |  | **1.43** | **1.46** | **0.25** | **0.10** | **2e-3 (MWUS)** |
|  |  |  | **Energy [kBT]** |  | **10.33** | **11.07** | **2.93** | **1.19** | **1e-3 (MWUS)** |
|  |  | **female** | f0 [Hz] | 4 | 244.35 | 241.15 | 54.24 | 27.12 | 0.0862 |
|  |  |  | Q |  | 2.16 | 2.27 | 0.59 | 0.29 | 0.823 |
|  |  |  | **Energy [kBT]** |  | **8.8** | **8.79** | **3.05** | **1.52** | **0.0122** |
|  | **day25** | **male** | **f0 [Hz]** | **19** | **274.37** | **274.50** | **48.26** | **11.07** | **P = <0.001 (MWUS)** |
|  |  |  | **Q** |  | **4.87** | **4.50** | **2.14** | **0.49** | **P = <0.001 (MWUS)** |
|  |  |  | **Energy [kBT]** |  | **33.31** | **29.47** | **13.70** | **3.14** | **0.012 (MWUS)** |
|  |  | female | f0 [Hz] | 17 | 212.49 | 211.40 | 29.87 | 7.24 | 0.098 |
|  |  |  | Q |  | 2.79 | 2.29 | 1.28 | 0.31 | 0.121 |
|  |  |  | Energy [kBT] |  | 18.70 | 18.38 | 8.10 | 1.96 | 0.428 |
|  | **day50** | **male** | **f0 [Hz]** | **16** | **264.65** | **260.12** | **45.86** | **11.46** | **0.008 (MWUS)** |
|  |  |  | Q |  | 2.32 | 1.99 | 1.44 | 0.36 | 0.234 |
|  |  |  | Energy [kBT] |  | 19.48 | 17.87 | 7.74 | 1.94 | 0.162 |
|  |  | **female** | f0 [Hz] | 20 | 220.15 | 230.52 | 35.89 | 8.02 | 0.0952 |
|  |  |  | Q |  | 1.83 | 1.69 | 0.67 | 0.15 | 0.124 |
|  |  |  | **Energy [kBT]** |  | **10.35** | **10.33** | **3.84** | **0.86** | **P = <0.001 (MWUS)** |
|  | **day60** | **male** | **f0 [Hz]** | **11** | **401.72** | **366.26** | **129.87** | **39.16** | **P = <0.001 (MWUS)** |
|  |  |  | **Q** |  | **1.44** | **1.28** | **0.38** | **0.12** | **P = <0.001 (MWUS)** |
|  |  |  | **Energy [kBT]** |  | **7.53** | **7.68** | **4.51** | **1.36** | **P = <0.001 (MWUS)** |
|  |  | **female** | **f0 [Hz]** | **12** | **243.38** | **234.37** | **29.08** | **8.39** | **4e-3 (MWUS)** |
|  |  |  | **Q** |  | **1.25** | **1.14** | **0.38** | **0.11** | **P = <0.001 (MWUS)** |
|  |  |  | **Energy [kBT]** |  | **5.60** | **5.15** | **2.03** | **0.59** | **P = <0.001 (MWUS)** |
|  | **day70** | **male** | **f0 [Hz]** | **18** | **442.85** | **425.92** | **169.67** | **39.99** | **P = <0.001 (MWUS)** |
|  |  |  | **Q** |  | **1.09** | **1.04** | **0.32** | **0.08** | **P = <0.001 (MWUS)** |
|  |  |  | **Energy [kBT]** |  | **2.95** | **2.20** | **2.30** | **0.54** | **P = <0.001 (MWUS)** |
|  |  | **female** | **f0 [Hz]** | **17** | **439.19** | **359.96** | **195.84** | **47.50** | **P = <0.001 (MWUS)** |
|  |  |  | **Q** |  | **1.45** | **1.39** | **0.53** | **0.13** | **3e-3 (MWUS)** |
|  |  |  | **Energy [kBT]** |  | **2.60** | **2.01** | **2.74** | **0.66** | **P = <0.001 (MWUS)** |
| OregonR | day1 | female | f0 [Hz] | 9 | 150.55 | 149.80 | 16.97 | 5.66 | -- |
|  |  |  | Q |  | 0.85 | 0.88 | 0.16 | 0.05 | -- |
|  |  |  | Energy [kBT] |  | 6.66 | 5.87 | 1.67 | 0.56 | -- |
|  | day5 | female | f0 [Hz] | 8 | 147.30 | 139.40 | 29.16 | 10.31 | 0.78 |
|  |  |  | Q |  | 0.80 | 0.80 | 0.17 | 0.06 | 0.52 |
|  |  |  | Energy [kBT] |  | 5.13 | 4.46 | 2.03 | 0.72 | 0.061 |
|  | day25 | female | f0 [Hz] | 5 | 164.01 | 160.66 | 69.37 | 31.02 | 0.79 |
|  |  |  | Q |  | 0.99 | 0.92 | 0.44 | 0.20 | 0.594 |
|  |  |  | Energy [kBT] |  | 7.14 | 5.30 | 3.58 | 1.60 | 0.732 |
|  | **day50** | **female** | **f0 [Hz]** | **10** | **481.62** | **544.13** | **201.75** | **63.80** | **4e-3 (MWUS)** |
|  |  |  | Q |  | 0.70 | 0.72 | 0.19 | 0.06 | 0.0723 |
|  |  |  | **Energy [kBT]** |  | **1.14** | **0.76** | **1.19** | **0.38** | **P = <0.001 (MWUS)** |
| CantonS (G) | day1 | male | f0 [Hz] | 10 | 242.89 | 244.99 | 35.96 | 11.37 | -- |
|  |  |  | Q |  | 1.66 | 1.65 | 0.42 | 0.13 | -- |
|  |  |  | Energy [kBT] |  | 14.34 | 13.84 | 3.10 | 0.98 | -- |
|  |  | female | f0 [Hz] | 11 | 235.35 | 235.15 | 35.46 | 10.69 | -- |
|  |  |  | Q |  | 1.62 | 1.69 | 0.38 | 0.11 | -- |
|  |  |  | Energy [kBT] |  | 11.22 | 11.65 | 3.32 | 1.00 | -- |
|  | **day5** | **male** | f0 [Hz] | 12 | 268.96 | 273.22 | 28.19 | 8.14 | 0.071 |
|  |  |  | Q |  | 1.53 | 1.57 | 0.39 | 0.11 | 0.452 |
|  |  |  | **Energy [kBT]** |  | **9.27** | **9.34** | **2.46** | **0.71** | **3.64E-04** |
|  |  | **female** | f0 [Hz] | 9 | 264.02 | 253.02 | 42.16 | 14.05 | 0.116 |
|  |  |  | Q |  | 1.63 | 1.69 | 0.40 | 0.13 | 0.946 |
|  |  |  | **Energy [kBT]** |  | **7.41** | **7.88** | **3.24** | **1.08** | **0.0188** |
|  | day25 | male | f0 [Hz] | 13 | 263.78 | 264.93 | 36.44 | 10.11 | 0.185 |
|  |  |  | Q |  | 1.69 | 1.52 | 0.52 | 0.14 | 0.905 |
|  |  |  | Energy [kBT] |  | 12.11 | 10.57 | 4.58 | 1.27 | 0.201 |
|  |  | female | f0 [Hz] | 12 | 253.57 | 256.22 | 32.53 | 9.39 | 0.213 |
|  |  |  | Q |  | 2.21 | 1.81 | 1.10 | 0.32 | 0.103 |
|  |  |  | Energy [kBT] |  | 10.73 | 9.15 | 4.39 | 1.27 | 0.765 |
|  | **day50** | **male** | **f0 [Hz]** | **12** | **370.57** | **342.20** | **88.07** | **25.42** | **3.63E-04** |
|  |  |  | Q |  | 1.37 | 1.22 | 0.47 | 0.14 | 0.142 |
|  |  |  | **Energy [kBT]** |  | **8.22** | **9.15** | **3.72** | **1.08** | **5.15E-04** |
|  |  | **female** | **f0 [Hz]** | **11** | **298.22** | **292.68** | **34.15** | **10.30** | **4.05E-04** |
|  |  |  | Q |  | 1.56 | 1.51 | 0.42 | 0.13 | 0.751 |
|  |  |  | Energy [kBT] |  | 8.28 | 6.40 | 3.38 | 1.02 | 0.0528 |
|  | **day68** | **male** | **f0 [Hz]** | **5** | **470.63** | **428.17** | **144.02** | **64.41** | **6e-3 (MWUS)** |
|  |  |  | Q |  | 1.18 | 1.05 | 0.43 | 0.19 | 0.0608 |
|  |  |  | **Energy [kBT]** |  | **4.75** | **3.99** | **2.80** | **1.25** | **6.07E-05** |
|  |  | **female** | **f0 [Hz]** | **9** | **355.52** | **330.97** | **95.74** | **31.91** | **P = <0.001 (MWUS)** |
|  |  |  | Q |  | 2.17 | 2.06 | 0.95 | 0.32 | 0.224 |
|  |  |  | **Energy [kBT]** |  | **6.58** | **6.46** | **3.50** | **1.17** | **7.05E-03** |
|  | **day70** | **male** | **f0 [Hz]** | **8** | **590.29** | **588.41** | **167.25** | **59.13** | **P = <0.001 (MWUS)** |
|  |  |  | Q |  | 1.31 | 1.21 | 0.35 | 0.12 | 0.0797 |
|  |  |  | **Energy [kBT]** |  | **2.77** | **2.77** | **2.18** | **0.77** | **1.34E-07** |
|  |  | **female** | **f0 [Hz]** | **13** | **395.09** | **410.39** | **49.42** | **13.71** | **P = <0.001 (MWUS)** |
|  |  |  | Q |  | 1.60 | 1.69 | 0.47 | 0.13 | 0.922 |
|  |  |  | **Energy [kBT]** |  | **3.79** | **3.77** | **1.19** | **0.33** | **P = <0.001 (MWUS)** |
| CantonS (K) | day1 | male | f0 [Hz] | 12 | 256.60 | 255.85 | 26.09 | 7.53 | -- |
|  |  |  | Q |  | 1.53 | 1.64 | 0.27 | 0.08 | -- |
|  |  |  | Energy [kBT] |  | 10.59 | 11.51 | 2.88 | 0.83 | -- |
|  |  | female | f0 [Hz] | 10 | 213.18 | 213.91 | 34.64 | 10.95 | -- |
|  |  |  | Q |  | 1.41 | 1.33 | 0.46 | 0.14 | -- |
|  |  |  | Energy [kBT] |  | 9.05 | 9.45 | 2.51 | 0.79 | -- |
|  | **day5** | **male** | **f0 [Hz]** | **11** | **293.26** | **298.83** | **40.33** | **12.16** | **0.0163** |
|  |  |  | Q |  | 1.81 | 1.58 | 0.65 | 0.20 | 0.442 |
|  |  |  | **Energy [kBT]** |  | **8.03** | **7.62** | **2.36** | **0.71** | **0.0309** |
|  |  | female | f0 [Hz] | 10 | 252.48 | 234.85 | 56.75 | 17.94 | 0.054 |
|  |  |  | Q |  | 1.35 | 1.31 | 0.25 | 0.08 | 0.752 |
|  |  |  | **Energy [kBT]** |  | **5.88** | **5.61** | **1.82** | **0.57** | **4.63E-03** |
|  | **day25** | **male** | **f0 [Hz]** | **16** | **292.96** | **301.76** | **36.17** | **9.04** | **6.68E-03** |
|  |  |  | Q |  | 1.55 | 1.54 | 0.45 | 0.11 | 0.879 |
|  |  |  | **Energy [kBT]** |  | **6.48** | **6.77** | **2.58** | **0.65** | **5.16E-04** |
|  |  | **female** | **f0 [Hz]** | **11** | **288.89** | **288.09** | **10.98** | **3.31** | **P = <0.001 (MWUS)** |
|  |  |  | Q |  | 1.72 | 1.68 | 0.32 | 0.10 | 0.0785 |
|  |  |  | **Energy [kBT]** |  | **6.45** | **6.41** | **1.55** | **0.47** | **0.005 (MWUS)** |
|  | **day50** | **male** | **f0 [Hz]** | **10** | **368.92** | **367.62** | **32.31** | **9.74** | **4.60E-09** |
|  |  |  | **Q** |  | **1.77** | **1.89** | **0.58** | **0.18** | **0.044** |
|  |  |  | **Energy [kBT]** |  | **3.88** | **3.99** | **1.41** | **0.43** | **P = <0.001 (MWUS)** |
|  |  | **female** | **f0 [Hz]** | **12** | **360.80** | **347.37** | **43.22** | **12.48** | **3.07E-08** |
|  |  |  | **Q** |  | **2.29** | **1.95** | **0.88** | **0.25** | **9.19E-03** |
|  |  |  | **Energy [kBT]** |  | **4.05** | **4.15** | **0.98** | **0.28** | **1e-3 (MWUS)** |
|  | **day68** | **male** | **f0 [Hz]** | **7** | **377.56** | **388.55** | **35.42** | **13.39** | **1.44E-07** |
|  |  |  | **Q** |  | **1.95** | **2.11** | **0.46** | **0.17** | **0.0202** |
|  |  |  | **Energy [kBT]** |  | **3.92** | **3.99** | **1.12** | **0.42** | **2.04E-05** |
|  | **day60** | **female** | **f0 [Hz]** | **7** | **366.36** | **370.04** | **24.75** | **9.35** | **4.96E-08** |
|  |  |  | **Q** |  | **1.84** | **1.92** | **0.26** | **0.10** | **0.041** |
|  |  |  | **Energy [kBT]** |  | **3.60** | **3.35** | **1.06** | **0.40** | **4e-3 (MWUS)** |
|  | **day70** | **male** | **f0 [Hz]** | **4** | **353.80** | **363.42** | **22.19** | **11.09** | **1.09E-05** |
|  |  |  | **Q** |  | **1.45** | **1.51** | **0.71** | **0.36** | **0.952** |
|  |  |  | **Energy [kBT]** |  | **3.81** | **4.33** | **2.04** | **1.02** | **7.16E-04** |
