# Supplementary material for: Homeostatic maintenance and age-related functional decline in the Drosophila ear
Source: Sci Rep. 2020 May 4;10:7431. doi: 10.1038/s41598-020-64498-z (PMC7198581; doi:10.1038/s41598-020-64498-z)
Supplement: Supplementary file 7 — Supplementary Table 6. [file 41598_2020_64498_MOESM7_ESM.docx]

| Genotype | sex | N | | Parameter | Mean | | Median | | StandardDev | | StandardErr | | p-value |
| --- | --- | --- | --- | --- | --- | --- | --- | --- | --- | --- | --- | --- | --- |
| Aef1 KD | male | 8 | (12) | f0 [Hz] | 253.04 | (247.24) | 251.87 | (236.40) | 28.65 | (42.66) | 10.13 | (12.86) | 0.743 |
|  |  |  |  | Q | 1.03 | (1.05) | 1.04 | (1.09) | 0.16 | (0.26) | 0.06 | (0.08) | 0.773 |
|  |  |  |  | Energy [kBT] | 5.71 | (7.27) | 5.48 | (7.27) | 1.64 | (1.76) | 0.58 | (0.53) | 0.0678 |
|  | female | 10 | (11) | f0 [Hz] | 297.99 | (303.57) | 305.76 | (309.35) | 37.22 | (48.00) | 11.77 | (13.86) | 0.767 |
|  |  |  |  | Q | 1.30 | (1.41) | 1.28 | (1.38) | 0.27 | (0.34) | 0.09 | (0.10) | 0.433 |
|  |  |  |  | Energy [kBT] | 6.28 | (5.80) | 5.89 | (6.09) | 1.72 | (1.88) | 0.55 | (0.54) | 0.545 |
| **amos KD** | **male** | 9 | (7) | f0 [Hz] | 237.28 | (260.08) | 220.17 | (242.45) | 49.25 | (39.17) | 16.42 | (14.80) | 0.334 |
|  |  |  |  | **Q** | **0.85** | **(1.18)** | **0.87** | **(1.09)** | **0.16** | **(0.30)** | **0.05** | **(0.11)** | **6e-3 [MWRST]** |
|  |  |  |  | **Energy [kBT]** | **3.97** | **(7.19)** | **4.62** | **(6.56)** | **1.54** | **(2.06)** | **0.51** | **(0.78)** | **3.05E-03** |
|  | **female** | 9 | (8) | **f0 [Hz]** | **218.82** | **(274.60)** | **229.84** | **(256.11)** | **47.75** | **(45.67)** | **15.92** | **(16.15)** | **0.0269** |
|  |  |  |  | **Q** | **0.91** | **(1.63)** | **0.78** | **(1.28)** | **0.35** | **(0.91)** | **0.12** | **(0.32)** | **0.03 [MWUS]** |
|  |  |  |  | **Energy [kBT]** | **1.95** | **(4.01)** | **2.03** | **(4.06)** | **0.55** | **(0.99)** | **0.18** | **(0.35)** | **7.38E-05** |
| **aop KD** | **male** | 6 | (11) | f0 [Hz] | 251.18 | (247.24) | 257.59 | (236.40) | 25.25 | (42.66) | 10.31 | (12.86) | 0.84 |
|  |  |  |  | Q | 0.90 | (1.05) | 0.83 | (1.09) | 0.28 | (0.26) | 0.11 | (0.08) | 0.276 |
|  |  |  |  | **Energy [kBT]** | **9.47** | **(7.27)** | **9.68** | **(7.27)** | **2.00** | **(1.76)** | **0.82** | **(0.53)** | **0.0326** |
|  | female | 6 | (11) | f0 [Hz] | 279.39 | (309.11) | 280.63 | (310.65) | 32.80 | (46.14) | 13.39 | (13.91) | 0.185 |
|  |  |  |  | Q | 1.81 | (1.47) | 1.11 | (1.38) | 1.56 | (0.30) | 0.64 | (0.09) | 0.393 |
|  |  |  |  | Energy [kBT] | 7.09 | (5.67) | 7.09 | (5.66) | 3.02 | (1.92) | 1.23 | (0.58) | 0.252 |
| **ara KD** | **male** | 8 | (10) | f0 [Hz] | 292.99 | (266.39) | 293.97 | (265.83) | 28.72 | (47.35) | 10.15 | (14.97) | 0.083 |
|  |  |  |  | Q | **1.67** | **(1.04)** | **1.62** | **(1.00)** | **0.23** | **(0.32)** | **0.08** | **(0.10)** | **2.53E-04** |
|  |  |  |  | Energy [kBT] | **12.05** | **(4.42)** | **12.27** | **(4.37)** | **3.83** | **(1.78)** | **1.35** | **(0.56)** | **3.88E-05** |
|  | **female** | 6 | (11) | f0 [Hz] | 259.80 | (282.12) | 267.75 | (275.95) | 24.28 | (32.67) | 9.91 | (9.85) | 0.165 |
|  |  |  |  | Q | 1.78 | (1.41) | 1.62 | (1.38) | 0.67 | (0.30) | 0.27 | (0.09) | 0.133 |
|  |  |  |  | **Energy [kBT]** | **9.52** | **(4.48)** | **9.46** | **(3.93)** | **2.94** | **(1.53)** | **1.20** | **(0.46)** | **3e-3 [MWRST]** |
| ato KD | male | 12 | (11) | f0 [Hz] | 285.15 | (247.24) | 280.16 | (236.40) | 59.38 | (42.66) | 17.14 | (12.86) | 0.0958 |
|  |  |  |  | Q | 1.17 | (1.05) | 1.01 | (1.09) | 0.46 | (0.26) | 0.13 | (0.08) | 0.829 |
|  |  |  |  | Energy [kBT] | 6.38 | (7.27) | 5.63 | (7.27) | 2.71 | (1.76) | 0.78 | (0.53) | 0.364 |
|  | female | 10 | (12) | f0 [Hz] | 273.07 | (303.57) | 279.71 | (309.35) | 36.26 | (48.00) | 11.47 | (13.86) | 0.114 |
|  |  |  |  | Q | 1.24 | (1.41) | 1.21 | (1.38) | 0.24 | (0.34) | 0.08 | (0.10) | 0.194 |
|  |  |  |  | Energy [kBT] | 4.50 | (5.80) | 4.39 | (6.09) | 1.06 | (1.88) | 0.34 | (0.54) | 0.0669 |
| **ct KD** | **male** | 8 | (9) | f0 [Hz] | 297.48 | (235.00) | 266.82 | (223.77) | 125.48 | (28.32) | 44.36 | (9.44) | 0.163 |
|  |  |  |  | Q | 1.21 | (0.99) | 1.06 | (0.97) | 0.38 | (0.25) | 0.13 | (0.08) | 0.16 |
|  |  |  |  | **Energy [kBT]** | **4.78** | **(7.63)** | **5.02** | **(7.66)** | **2.13** | **(1.75)** | **0.75** | **(0.58)** | **8.38E-03** |
|  | female | 8 | (12) | f0 [Hz] | 290.15 | (303.57) | 296.88 | (309.35) | 39.29 | (48.00) | 13.89 | (13.86) | 0.52 |
|  |  |  |  | Q | 1.23 | (1.41) | 1.23 | (1.38) | 0.13 | (0.34) | 0.05 | (0.10) | 0.17 |
|  |  |  |  | Energy [kBT] | 4.76 | (5.80) | 4.42 | (6.09) | 1.90 | (1.88) | 0.67 | (0.54) | 0.245 |
| **Dhc98D KD** | **female** | 10 | (18) | f0 [Hz] | 347.14 | (276.94) | 282.40 | (284.77) | 183.24 | (59.16) | 57.94 | (13.94) | 0.719 |
|  |  |  |  | **Q** | **0.73** | **(1.25)** | **0.73** | **(1.33)** | **0.23** | **(0.40)** | **0.07** | **(0.09)** | **9.85E-04** |
|  |  |  |  | **Energy [kBT]** | **1.85** | **(5.19)** | **1.08** | **(4.75)** | **1.91** | **(1.87)** | **0.60** | **(0.44)** | **1.26E-04** |
| **gl KD** | **male** | 7 | [7) | **f0 [Hz]** | **310.56** | **(257.56)** | **326.21** | **(263.27)** | **34.71** | **(45.77)** | **13.12** | **(16.18)** | **0.0469** |
|  |  |  |  | Q | 1.03 | (0.91) | 1.06 | (0.96) | 0.18 | (0.19) | 0.07 | (0.07) | 0.164 |
|  |  |  |  | Energy [kBT] | 4.35 | (3.93) | 4.21 | (3.81) | 1.28 | (1.40) | 0.48 | (0.50) | 0.619 |
|  | **female** | 5 | [7) | f0 [Hz] | 278.87 | (283.93) | 286.62 | (270.60) | 20.59 | (41.26) | 9.21 | (15.60) | 0.807 |
|  |  |  |  | Q | 1.07 | (1.40) | 1.06 | (1.32) | 0.20 | (0.35) | 0.09 | (0.13) | 0.0887 |
|  |  |  |  | **Energy [kBT]** | **2.20** | **(4.50)** | **2.27** | **(3.88)** | **0.83** | **(1.61)** | **0.37** | **(0.61)** | **0.0157** |
| **lola KD** | **male** | 5 | [9) | **f0 [Hz]** | **323.02** | **(235.00)** | **332.34** | **(223.77)** | **29.60** | **(28.32)** | **13.24** | **(9.44)** | **1.39E-04** |
|  |  |  |  | Q | 1.00 | (0.99) | 1.09 | (0.97) | 0.21 | (0.25) | 0.09 | (0.08) | 0.924 |
|  |  |  |  | **Energy [kBT]** | **5.33** | **(7.63)** | **5.25** | **(7.66)** | **1.80** | **(1.75)** | **0.80** | **(0.58)** | **0.0377** |
|  | **female** | 6 | [11) | f0 [Hz] | 324.22 | (309.11) | 322.01 | (310.65) | 24.36 | (46.14) | 9.94 | (13.91) | 0.471 |
|  |  |  |  | **Q** | **1.14** | **(1.47)** | **1.14** | **(1.38)** | **0.09** | **(0.30)** | **0.04** | **(0.09)** | **0.0217** |
|  |  |  |  | Energy [kBT] | 5.09 | (5.67) | 5.36 | (5.66) | 1.18 | (1.92) | 0.48 | (0.58) | 0.58 |
| **onecut KD** | **male** | 10 | [11) | **f0 [Hz]** | **499.10** | **(247.24)** | **440.20** | **(236.40)** | **146.93** | **(42.66)** | **46.46** | **(12.86)** | **P = <0.001 [MWRST]** |
|  |  |  |  | Q | 1.07 | (1.05) | 0.96 | (1.09) | 0.32 | (0.26) | 0.10 | (0.08) | 0.846 |
|  |  |  |  | **Energy [kBT]** | **2.63** | **(7.27)** | **2.35** | **(7.27)** | **1.51** | **(1.76)** | **0.48** | **(0.53)** | **3.55E-06** |
|  | **female** | 15 | [12) | **f0 [Hz]** | **420.61** | **(303.57)** | **407.05** | **(309.35)** | **37.42** | **(48.00)** | **9.66** | **(13.86)** | **1.81E-07** |
|  |  |  |  | **Q** | **0.88** | **(1.41)** | **0.90** | **(1.38)** | **0.16** | **(0.34)** | **0.04** | **(0.10)** | **1.55E-05** |
|  |  |  |  | **Energy [kBT]** | **1.50** | **(5.80)** | **1.58** | **(6.09)** | **0.54** | **(1.88)** | **0.14** | **(0.54)** | **P = <0.001 [MWRST]** |
| **Optix KD** | **male** | 10 | [11) | f0 [Hz] | 269.38 | (247.24) | 251.15 | (236.40) | 48.29 | (42.66) | 15.27 | (12.86) | 0.193 |
|  |  |  |  | **Q** | **1.45** | **(1.05)** | **1.34** | **(1.09)** | **0.33** | **(0.26)** | **0.10** | **(0.08)** | **5.14E-03** |
|  |  |  |  | Energy [kBT] | 7.30 | (7.27) | 7.39 | (7.27) | 1.94 | (1.76) | 0.61 | (0.53) | 0.971 |
|  | **female** | 16 | [12) | **f0 [Hz]** | **243.94** | **(303.57)** | **241.04** | **(309.35)** | **25.29** | **(48.00)** | **6.32** | **(13.86)** | **0.000237** |
|  |  |  |  | Q | 1.55 | (1.41) | 1.48 | (1.38) | 0.51 | (0.34) | 0.13 | (0.10) | 0.562 |
|  |  |  |  | **Energy [kBT]** | **8.06** | **(5.80)** | **7.47** | **(6.09)** | **2.30** | **(1.88)** | **0.58** | **(0.54)** | **0.022 [MWUS]** |
| **pnr KD** | **male** | 5 | [11) | **f0 [Hz]** | **314.09** | **(247.24)** | **319.14** | **(236.40)** | **37.71** | **(42.66)** | **16.87** | **(12.86)** | **9.54E-03** |
|  |  |  |  | Q | 1.28 | (1.05) | 1.21 | (1.09) | 0.30 | (0.26) | 0.13 | (0.08) | 0.137 |
|  |  |  |  | Energy [kBT] | 7.27 | (7.27) | 7.86 | (7.27) | 1.73 | (1.76) | 0.77 | (0.53) | 1 |
|  | female | 5 | [12) | f0 [Hz] | 282.33 | (303.57) | 291.46 | (309.35) | 16.19 | (48.00) | 7.24 | (13.86) | 0.357 |
|  |  |  |  | Q | 1.37 | (1.41) | 1.48 | (1.38) | 0.41 | (0.34) | 0.18 | (0.10) | 0.822 |
|  |  |  |  | Energy [kBT] | 6.56 | (5.80) | 6.59 | (6.09) | 2.05 | (1.88) | 0.92 | (0.54) | 0.468 |
| **Pph13 KD** | **male** | 10 | [8) | f0 [Hz] | 278.64 | (257.56) | 276.56 | (263.27) | 36.82 | (45.77) | 11.64 | (16.18) | 0.294 |
|  |  |  |  | **Q** | **1.46** | **(0.91)** | **1.49** | **(0.96)** | **0.41** | **(0.19)** | **0.13** | **(0.07)** | **3.08E-03** |
|  |  |  |  | **Energy [kBT]** | **6.28** | **(3.93)** | **6.33** | **(3.81)** | **2.46** | **(1.40)** | **0.78** | **(0.50)** | **0.0289** |
|  | female | 8 | [9) | f0 [Hz] | 297.15 | (282.57) | 298.02 | (270.60) | 45.70 | (36.45) | 16.16 | (12.15) | 0.476 |
|  |  |  |  | Q | 1.97 | (1.43) | 1.46 | (1.38) | 1.43 | (0.32) | 0.51 | (0.11) | 0.665 |
|  |  |  |  | Energy [kBT] | 6.64 | (4.52) | 6.11 | (3.88) | 2.54 | (1.70) | 0.90 | (0.57) | 0.0582 |
| Rfx KD | male | 6 | [11) | f0 [Hz] | 249.11 | (247.24) | 249.99 | (236.40) | 37.67 | (42.66) | 15.38 | (12.86) | 0.93 |
|  |  |  |  | Q | 1.09 | (1.05) | 0.94 | (1.09) | 0.31 | (0.26) | 0.13 | (0.08) | 0.761 |
|  |  |  |  | Energy [kBT] | 5.93 | (7.27) | 5.57 | (7.27) | 1.29 | (1.76) | 0.53 | (0.53) | 0.125 |
|  | female | 7 | [12) | f0 [Hz] | 289.88 | (303.57) | 283.49 | (309.35) | 41.19 | (48.00) | 15.57 | (13.86) | 0.537 |
|  |  |  |  | Q | 1.43 | (1.41) | 1.34 | (1.38) | 0.43 | (0.34) | 0.16 | (0.10) | 0.923 |
|  |  |  |  | Energy [kBT] | 6.01 | (5.80) | 5.45 | (6.09) | 2.69 | (1.88) | 1.02 | (0.54) | 0.842 |
| **run KD** | **male** | 9 | [9) | **f0 [Hz]** | **303.89** | **(235.00)** | **290.25** | **(223.77)** | **40.60** | **(28.32)** | **13.53** | **(9.44)** | **2.00E-03** |
|  |  |  |  | **Q** | **1.52** | **(0.99)** | **1.58** | **(0.97)** | **0.49** | **(0.25)** | **0.16** | **(0.08)** | **8.00E-03** |
|  |  |  |  | Energy [kBT] | 9.10 | (7.63) | 8.76 | (7.66) | 4.29 | (1.75) | 1.43 | (0.58) | 0.377 |
|  | female | 4 | [12) | f0 [Hz] | 286.85 | (303.57) | 295.09 | (309.35) | 40.44 | (48.00) | 20.22 | (13.86) | 0.543 |
|  |  |  |  | Q | 1.59 | (1.41) | 1.53 | (1.38) | 0.54 | (0.34) | 0.27 | (0.10) | 0.454 |
|  |  |  |  | Energy [kBT] | 6.63 | (5.80) | 5.81 | (6.09) | 3.08 | (1.88) | 1.54 | (0.54) | 0.52 |
| **Sox14 KD** | **male** | 5 | [11) | f0 [Hz] | 273.29 | (247.24) | 271.38 | (236.40) | 11.11 | (42.66) | 4.97 | (12.86) | 0.113 |
|  |  |  |  | Q | 1.22 | (1.05) | 1.26 | (1.09) | 0.13 | (0.26) | 0.06 | (0.08) | 0.187 |
|  |  |  |  | **Energy [kBT]** | **4.69** | **(7.27)** | **4.94** | **(7.27)** | **1.23** | **(1.76)** | **0.55** | **(0.53)** | **0.0109** |
|  | female | 7 | [12) | f0 [Hz] | 277.09 | (303.57) | 268.76 | (309.35) | 55.07 | (48.00) | 20.82 | (13.86) | 0.287 |
|  |  |  |  | Q | 1.36 | (1.41) | 1.18 | (1.38) | 0.28 | (0.34) | 0.11 | (0.10) | 0.744 |
|  |  |  |  | Energy [kBT] | 4.55 | (5.80) | 4.92 | (6.09) | 1.64 | (1.88) | 0.62 | (0.54) | 0.164 |
| srp KD | male | 8 | [11) | f0 [Hz] | 284.02 | (247.24) | 287.25 | (236.40) | 31.45 | (42.66) | 11.12 | (12.86) | 0.0551 |
|  |  |  |  | Q | 1.12 | (1.05) | 0.99 | (1.09) | 0.34 | (0.26) | 0.12 | (0.08) | 0.634 |
|  |  |  |  | Energy [kBT] | 6.32 | (7.27) | 6.37 | (7.27) | 1.86 | (1.76) | 0.66 | (0.53) | 0.271 |
|  | female | 6 | [12) | f0 [Hz] | 293.36 | (303.57) | 296.23 | (309.35) | 14.49 | (48.00) | 5.92 | (13.86) | 0.622 |
|  |  |  |  | Q | 1.23 | (1.41) | 1.18 | (1.38) | 0.28 | (0.34) | 0.11 | (0.10) | 0.282 |
|  |  |  |  | Energy [kBT] | 5.91 | (5.80) | 5.35 | (6.09) | 1.00 | (1.88) | 0.41 | (0.54) | 0.963 |
| **Stat92E KD** | male | 9 | [11) | f0 [Hz] | 286.36 | (247.24) | 300.36 | (236.40) | 48.83 | (42.66) | 16.28 | (12.86) | 0.0718 |
|  |  |  |  | Q | 0.98 | (1.05) | 0.91 | (1.09) | 0.19 | (0.26) | 0.06 | (0.08) | 0.504 |
|  |  |  |  | Energy [kBT] | 5.87 | (7.27) | 6.08 | (7.27) | 2.18 | (1.76) | 0.73 | (0.53) | 0.128 |
|  | **female** | 7 | [12) | f0 [Hz] | 302.75 | (303.57) | 301.52 | (309.35) | 20.59 | (48.00) | 7.78 | (13.86) | 0.967 |
|  |  |  |  | **Q** | **1.11** | **(1.41)** | **1.11** | **(1.38)** | **0.18** | **(0.34)** | **0.07** | **(0.10)** | **0.0484** |
|  |  |  |  | Energy [kBT] | 4.96 | (5.80) | 4.82 | (6.09) | 0.82 | (1.88) | 0.31 | (0.54) | 0.281 |
| Tbp KD | male | 10 | [7) | f0 [Hz] | 254.29 | (260.08) | 234.91 | (242.45) | 58.22 | (39.17) | 18.41 | (14.80) | 0.823 |
|  |  |  |  | Q | 1.19 | (1.18) | 1.09 | (1.09) | 0.35 | (0.30) | 0.11 | (0.11) | 0.98 |
|  |  |  |  | Energy [kBT] | 5.82 | (7.19) | 5.47 | (6.56) | 2.55 | (2.06) | 0.81 | (0.78) | 0.256 |
|  | female | 9 | [8) | f0 [Hz] | 246.73 | (274.60) | 248.12 | (256.11) | 21.39 | (45.67) | 7.13 | (16.15) | 0.121 |
|  |  |  |  | Q | 1.31 | (1.63) | 1.23 | (1.28) | 0.44 | (0.91) | 0.15 | (0.32) | 0.532 |
|  |  |  |  | Energy [kBT] | 4.09 | (4.01) | 3.75 | (4.06) | 1.70 | (0.99) | 0.57 | (0.35) | 0.905 |
| **wor KD** | **male** | 7 | [8) | f0 [Hz] | 297.67 | (257.56) | 300.41 | (263.27) | 36.91 | (45.77) | 13.95 | (16.18) | 0.0873 |
|  |  |  |  | **Q** | **1.77** | **(0.91)** | **1.66** | **(0.96)** | **0.35** | **(0.19)** | **0.13** | **(0.07)** | **P= <0.001 [MWRST]** |
|  |  |  |  | **Energy [kBT]** | **12.36** | **(3.93)** | **10.74** | **(3.81)** | **4.40** | **(1.40)** | **1.66** | **(0.50)** | **P = <0.001 [MWRST]** |
|  | **female** | 14 | [15) | f0 [Hz] | 266.28 | (277.04) | 259.42 | (275.95) | 37.25 | (30.82) | 9.95 | (7.96) | 0.403 |
|  |  |  |  | **Q** | **2.59** | **(1.41)** | **1.99** | **(1.38)** | **1.65** | **(0.31)** | **0.44** | **(0.08)** | **P = <0.001 [MWRST]** |
|  |  |  |  | **Energy [kBT]** | **9.75** | **(4.31)** | **8.66** | **(3.88)** | **3.87** | **(1.48)** | **1.04** | **(0.38)** | **P = <0.001 [MWRST]** |
| **amos OE [30d]** | **male** | 4 | [6) | **f0 [Hz]** | **279.72** | **(419.64)** | **286.13** | **(399.99)** | **71.42** | **(98.17)** | **35.71** | **(40.08)** | **0.041** |
|  |  |  |  | Q | 1.08 | (0.88) | 1.01 | (0.84) | 0.35 | (0.29) | 0.17 | (0.12) | 0.365 |
|  |  |  |  | **Energy [kBT]** | **8.80** | **(1.20)** | **8.95** | **(1.05)** | **3.60** | **(0.59)** | **1.80** | **(0.24)** | **P = 0.01 [MWRST]** |
|  | **female** | 9 | [10) | f0 [Hz] | 304.84 | (336.28) | 298.36 | (326.49) | 46.85 | (45.84) | 15.62 | (14.50) | 0.158 |
|  |  |  |  | **Q** | **1.25** | **(0.93)** | **1.18** | **(0.93)** | **0.34** | **(0.22)** | **0.11** | **(0.07)** | **0.0253** |
|  |  |  |  | **Energy [kBT]** | **7.03** | **(1.13)** | **6.91** | **(1.11)** | **2.55** | **(0.71)** | **0.85** | **(0.22)** | **P = <0.001 [MWRST]** |
| **Optix OE [15d]** | **male** | 4 | [3) | **f0 [Hz]** | **591.51** | **(320.86)** | **606.59** | **(313.55)** | **115.18** | **(21.63)** | **57.59** | **(12.49)** | **0.0111** |
|  |  |  |  | Q | 0.94 | (1.23) | 0.91 | (1.26) | 0.20 | (0.55) | 0.10 | (0.32) | 0.629 |
|  |  |  |  | **Energy [kBT]** | **0.43** | **(5.88)** | **0.44** | **(6.85)** | **0.25** | **(3.10)** | **0.12** | **(1.79)** | **0.0152** |
|  | **female** | 5 | [8) | **f0 [Hz]** | **355.45** | **(278.15)** | **386.37** | **(280.29)** | **62.44** | **(21.54)** | **27.92** | **(7.62)** | **7.38E-03** |
|  |  |  |  | **Q** | **1.21** | **(1.76)** | **1.28** | **(1.75)** | **0.39** | **(0.40)** | **0.18** | **(0.14)** | **0.0346** |
|  |  |  |  | **Energy [kBT]** | **1.87** | **(6.94)** | **2.26** | **(6.50)** | **1.12** | **(3.16)** | **0.50** | **(1.12)** | **5.83E-03** |
| **Optix OE [30d]** | **female** | 6 | [10) | **f0 [Hz]** | **449.41** | **(336.28)** | **465.23** | **(326.49)** | **36.54** | **(45.84)** | **14.92** | **(14.50)** | **1.55E-04** |
|  |  |  |  | **Q** | **1.27** | **(0.93)** | **1.24** | **(0.93)** | **0.24** | **(0.22)** | **0.10** | **(0.07)** | **0.0125** |
|  |  |  |  | Energy [kBT] | 1.22 | (1.13) | 1.20 | (1.11) | 0.65 | (0.71) | 0.26 | (0.22) | 0.807 |
| wor OE [15d] | male | 8 | [10) | f0 [Hz] | 304.60 | (266.39) | 296.64 | (265.83) | 26.10 | (47.35) | 9.23 | (14.97) | 0.0582 |
|  |  |  |  | Q | 1.27 | (1.04) | 1.23 | (1.00) | 0.24 | (0.32) | 0.09 | (0.10) | 0.116 |
|  |  |  |  | Energy [kBT] | 5.77 | (4.42) | 5.00 | (4.37) | 1.87 | (1.78) | 0.66 | (0.56) | 0.139 |
|  | female | 6 | [15) | f0 [Hz] | 261.36 | (277.04) | 236.75 | (275.95) | 60.68 | (30.82) | 24.77 | (7.96) | 0.437 |
|  |  |  |  | Q | 1.19 | (1.41) | 1.07 | (1.38) | 0.45 | (0.31) | 0.18 | (0.08) | 0.208 |
|  |  |  |  | Energy [kBT] | 4.46 | (4.31) | 4.00 | (3.88) | 1.15 | (1.48) | 0.47 | (0.38) | 0.669 |
| **gl OE [30d]** | male | 4 | [6) | f0 [Hz] | 388.92 | (419.64) | 404.10 | (399.99) | 58.65 | (98.17) | 29.33 | (40.08) | 0.593 |
|  |  |  |  | Q | 0.69 | (0.88) | 0.71 | (0.84) | 0.15 | (0.29) | 0.07 | (0.12) | 0.255 |
|  |  |  |  | Energy [kBT] | 0.96 | (1.20) | 0.99 | (1.05) | 0.26 | (0.59) | 0.13 | (0.24) | 0.458 |
|  | **female** | 4 | [7) | **f0 [Hz]** | **214.44** | **(326.72)** | **211.58** | **(321.16)** | **16.30** | **(49.25)** | **8.15** | **(18.61)** | **1.88E-03** |
|  |  |  |  | **Q** | **0.51** | **(0.95)** | **0.48** | **(0.96)** | **0.10** | **(0.19)** | **0.05** | **(0.07)** | **6.00E-03** |
|  |  |  |  | Energy [kBT] | 0.95 | (1.36) | 1.07 | (1.49) | 0.28 | (0.74) | 0.14 | (0.28) | 0.323 |
| **ato OE** | **female** | 9 | [10) | f0 [Hz] | 299.31 | (336.28) | 290.41 | (326.49) | 115.45 | (45.84) | 38.48 | (14.50) | 0.54 |
|  |  |  |  | Q | 1.34 | (0.93) | 0.99 | (0.93) | 0.81 | (0.22) | 0.27 | (0.07) | 0.348 |
|  |  |  |  | **Energy [kBT]** | **4.36** | **(1.13)** | **4.56** | **(1.11)** | **1.71** | **(0.71)** | **0.57** | **(0.22)** | **P = <0.001 [MWRST]** |
